# Evaluation of Chongqing Tuo Tea at Different Grades: An Integrated Approach by Artificial and Intelligent Sensory, Non-Volatile, and Volatile Compounds Analysis

**DOI:** 10.3390/foods13060865

**Published:** 2024-03-13

**Authors:** Yiwen Miao, Lilei Wang, Fei Bai, Shuting Zheng, Jingna Yan, Hao Wei, Qing Meng, Huarong Tong

**Affiliations:** 1 College of Food Science, Southwest University, Beibei District, Chongqing 400715, China; mywfood@email.swu.edu.cn (Y.M.); llwang309@163.com (L.W.); feifei111@email.swu.edu.cn (F.B.); zhengshuting978@163.com (S.Z.); weihao17660713491@163.com (H.W.); mengqing2015@swu.edu.cn (Q.M.); 2 School of Modern Agriculture, Yibin Vocational and Technical College, Yibin 644000, China; yan1202na@163.com

**Keywords:** Tuo tea, quality grades, artificial and intelligent sensory, aroma, taste

## Abstract

This study aims to investigate the relationship between the grades of Tuo tea and the quality of compounds. A combination of artificial sensory evaluation, intelligent sensory technologies (electronic nose and electronic tongue), gas chromatography–mass spectrometry (GC-MS), high-performance liquid chromatography (HPLC), chemical–physical analysis, and multivariate statistical analysis were employed to examine the differences among three grades of Tuo tea (SG, 1G, and 2G). The results of artificial sensory evaluation, electronic tongue, and electronic nose revealed that the aroma and taste of different grades of Tuo tea varied greatly. A total of 112 volatile compounds and 44 non-volatile compounds were identified. In order to elucidate the key components that cause differences in the quality of Tuo tea, 2 partial least squares discriminant analysis (PLS-DA) models with excellent parameters (volatile, R^2^Y = 0.999 and Q^2^ = 0.996; non-volatile, R^2^Y = 0.992 and Q^2^ = 0.972) were established. A total of 80 key differential volatile compounds were identified with the double selection criterion of variable importance in projection (VIP) greater than 1 and *p* < 0.05. Among these, 43 compounds with OAV > 1 were further identified as the odor-active compounds in all three grades of Tuo. Moreover, 22 key non-volatile compounds that contribute to the quality differences have been screened out. This investigation implied that the volatile and non-volatile compounds of Tuo tea could serve as indicators of its quality. The results provided a new approach to distinguish the grades of Tuo tea.

## 1. Introduction

Tea is one of the most widely consumed non-alcoholic beverages in the world and is prized for its unique flavor and health benefits [1]. Tuo tea is a type of reprocessed product, with a bowl shape and an aged aroma, including Yunnan Tuo tea and Chongqing Tuo tea. Yunnan Tuo tea is mainly processed from Yunnan large leaf sun-dried green tea, while Chongqing Tuo tea is processed by blending Yunnan large leaf sun-dried green tea with hot-air dried and roasted green tea from small and medium-sized leaf varieties. The earliest production process of Chongqing Tuo tea emerged in the 1950s. According to the local standard “DB 50/T 1099-2021 Chongqing Tuo Tea Processing Technical Regulations”, the processing procedure included the refining of raw green tea (sun-dried, roasted, and hot-air dried), blending, weighing, steaming, pressing, shaping, and drying [2,3,4]. On this basis, varieties, processing techniques, and geographical origins have been reported to contribute to the formation of quality differences in Tuo tea [5]. However, little research has been available on the differences in quality grades of Tuo tea. At present, according to the artificial sensory evaluation, Tuo tea is artificially divided into three grades (special grade (SG), first grade (1G), and second grade (2G) [6]. Artificial sensory evaluation makes it difficult to clarify the correlation between composition and quality grades. Therefore, the utilization of instrumental analysis techniques is crucial for delineating the compositional disparities among Tuo tea of varying grades.

Complex metabolites provide a material basis for the diversity of tea flavor [7]. In general, higher-grade tea exhibits superior flavor characteristics, and consumers tend to prefer high-quality tea [8]. Hence, the establishment of a rational, objective, and efficient evaluation method for discriminating various grades of Chongqing Tuo tea not only facilitates rapid grade identification but also meets the interests of consumers. However, the research on Tuo tea has predominantly centered on documenting its sensory attributes and chemical composition so far. For example, Chang et al. studied Yunling, Xiaguan, and Shancheng Tuo tea and found that different types of Tuo tea were associated with non-volatile compounds in terms of sensory quality [5]. During microbial fermentation, the contents of tea polyphenols, caffeine, theanine, and free amino acids in Xiaguan Tuo tea underwent changes [9]. Su et al. declared that the content of volatile oil in Tuo tea accounts for approximately 0.0112% of dry weight [4]. Luo et al. found that different drying methods had a significant impact on the formation of aroma in Tuo tea [10]. Nevertheless, it is noteworthy that existing research has overlooked the dissimilarities in internal compounds among various grades of Chongqing Tuo tea, and the association between sensory quality and internal compounds of different grades of Chongqing Tuo tea remains ambiguous.

Prior research has indicated a correlation between the tea grade and its metabolites. For instance, linalool, benzyl alcohol, and methyl salicylate were important contributors to the aroma of premium Keemun black tea [8]. Wang et al. found that 2-methylfuran, 2-ethylfuran, 2-methylidenecyclopentan-1-ol, mesityl oxide, 2-amylfuran, and d-limonene in Tieguanyin tea were negatively correlated with its grade [11]. A rapid and accurate chemometrics-assisted high-performance liquid chromatography (HPLC) method was developed in Xihu Longjing teas with different grades by Gu et al. [12]. Nevertheless, there has been limited attention given to the differences among different grades of Chongqing Tuo tea in terms of their impact on quality.

In this study, integration of artificial sensory evaluation, electronic tongue, electronic nose, HPLC and headspace-solid phase micro-extraction (HS-SPME) with gas chromatography–mass spectrometry (GC-MS), and chemical–physical analysis were applied to uncover the mystery between quality grades and metabolites. Subsequently, we utilized a range of multivariate statistical analyses such as heatmap, hierarchical cluster analysis (HCA), partial least squares discriminant analysis (PLS-DA), and variable importance in projection (VIP) to differentiate the distinct grades of Tuo tea and pinpoint potential differential metabolites. This work provided a new approach to differentiate between various grades of Chongqing Tuo tea and unveiled the chemical characteristics of Tuo tea, providing a deeper understanding of the underlying factors contributing to the variations in quality among the three grades of Chongqing Tuo tea.

## 2. Materials and Methods

### 2.1. Chemicals and Reagents

Pure water for tea brewing and extraction was provided by Wahaha Group Co., Ltd. (Hangzhou, China). The purities of all standards were above 98%. Eight types of catechins and 11 types of flavones and flavonol glycosides standards were purchased from Yuanye Bio-Technology Corporation (Shanghai, China). A mixed standards solution of 17 amino acids was purchased from the National Institute of Metrology (Beijing, China). The γ-aminobutyric acid (GABA), glutamine (Gln), asparagine (Asn), and tryptophan (Trp) standards were purchased from Adamas-beta Industrial Corporation (Shanghai, China). Standards of caffeine (CAF) and gallic acid (GA) were purchased from Chengdu Must Bio-technology Corporation (Chengdu, China). HPLC-grade methanol, acetonitrile, tetrahydrofuran, n-alkane mixture (C8~C32), and theanine (Thea) were purchased from Sigma Aldrich Trading Co., Ltd. (Shanghai, China). HPLC-grade acetic acid and ethyl decanoate were purchased from Shanghai Aladdin Biochemical Technology Co., Ltd. (Shanghai, China). Folin–Ciocalteu was purchased from Beijing Solebao Technology Co., Ltd. (Beijing, China). Na_2_CO_3_, DNFB, NaHCO_3_, NaAc, KH_2_PO_4_, NaOH, C_14_H_10_O, H_2_SO_4_, C_6_H_12_C_6_, and NaCl (analytical reagent) were purchased from Chengdu Kelong Chemical Reagent Factory (Chengdu, China).

### 2.2. Sample Preparation

Three samples of Tuo tea were from Chongqing Shanyu Yixiang Tea Industry Co., Ltd. (Chongqing, China). The samples were processed according to the Chongqing Tuo Tea Processing Technical Regulations (DB 50/T 1099-2021) in 2018, which included the refining of raw green tea (sun-dried, roasted, and hot-air dried), blending, weighing, steaming, pressing, shaping, and drying. At the same time, the produced Tuo tea materials were meticulously sealed in aluminum foil bags and stored at −40 °C for future analysis.

### 2.3. Sensory Evaluation

The sensory evaluation was conducted by a panel of three certified experts following the National Standard of China (GB/T23776-2018) [13]. Specifically, 3 g of Tuo tea were weighed and placed into an evaluation cup filled with 150 mL of boiling water. After 2 min steeping, the infusion was decanted, and a second infusion was repeated for 5 min. We employed a 100-point quality grading system, assigning weights of 20%, 10%, 30%, 35%, and 5% to dry tea appearance, liquor color, aroma, taste, and infused leaves, respectively. Before conducting the sensory evaluation, informed consent was obtained from all participants.

### 2.4. Electronic Nose Analysis

The electronic nose analysis was based on a previous study with slight modifications [14]. The gas detectors of the electronic nose system (Supplementary Materials Figure S1) consisted of ten metal oxide sensors, which were individually sensitive to different volatile organic compounds (Table 1). Typically, a precise amount of 2 g of tea powder was weighed and placed into a 40 mL headspace flask, which was then sealed with a silicone cap. The headspace flask was equilibrated in an electrically heated constant temperature blast drying oven at 60 °C for 60 min for the extraction. Subsequently, both the injection and gas-filling needle of the electronic nose (cNose, Shanghai Baosheng Industrial Development Co., Ltd., Shanghai, China) were simultaneously inserted into the headspace flask. Manual headspace injection was performed with an interval of 1 min between injections, a sample collection time of 90 s, an instrument cleaning time of 120 s, a gas flow rate of 0.8 L/min, and an operating ambient temperature maintained at 25 ± 2 °C.

### 2.5. Electronic Tongue Analysis

The taste of the tea infusion was determined using an electronic tongue apparatus (α-Astree II, Alpha MOS, Toulouse, France), consisting of a standard Ag/AgCl reference electrode and seven taste sensors, including AHS (sour), ANS (sweet), SCS (bitter), CTS (salty), NMS (umami), and PKS and CPS (comprehensive taste). The tea infusion was prepared according to the sensory evaluation, and the tea infusion brewed for the second time was tested. Before measurement, the sensors were cleaned for 30 s with ultrapure water as the cleaning solution. To measure the taste intensity, the sensor and reference probe were immersed in tea infusion and reference solution, respectively. The instrument parameters were set according to Wu et al. [15], with a data acquisition cycle of 1 s, an acquisition time of 120 s, and a stirring speed of 1 rad/s.

### 2.6. Extraction and Identification of Volatile Compounds

#### 2.6.1. HS-SPME Conditions

The extraction method for volatile compounds in Tuo tea was slightly modified from the approach developed by Kun et al. [16]. Simply, 1 g of tea powder, 2 g of NaCl, and 10 mL of pure water at 100 °C were added to a 40 mL headspace flask. An additional 25 μL of an internal standard solution was added to the mixture. A magnetic stirrer was then introduced into the bottle and sealed with a headspace cap. Next, the SPME fiber (50/30 µm DVB/CAR/PDMS, Supelco, Bellefonte, PA, USA) was inserted into the headspace flask containing the extraction mixture, pre-set at a temperature of 60 °C and stirred at a speed of 300 r/min for 5 min to reach equilibrium before undergoing an additional 55 min extraction process.

#### 2.6.2. Volatile Compounds Detection via GC-MS

The volatile compounds in the samples were analyzed using GC-MS (mod. QP 2010; Shimadzu, Tokyo, Japan), which was equipped with a DB-5MS capillary column (30 m × 0.25 mm, 0.25 µm, Agilent Technologies Inc., Santa Clara, CA, USA) and a flame ionization detector (FID). Helium with a purity of 99.999% was employed as the carrier gas at a linear velocity of 1.0 mL/min. The fiber performed thermal desorption at the injector port at 250 °C for 5 min in splitless mode. The temperature of FID was set to 250 °C. The temperature program for GC-MS was as follows: it was held at 40 °C for 2 min, then increased at a rate of 2 °C/min to 80 °C and held for 2 min. Subsequently, it was raised to 150 °C at a rate of 5 °C/min and held for 1 min, followed by an increase to 180 °C at a rate of 4 °C/min and held for 1 min. Finally, it was raised to 260 °C at a rate of 10 °C/min and held for 5 min. The conditions for MS analysis were as follows: the mass spectrometer was operational in the mode of electron ionization (EI), and EI source was used with an electron energy of 70 eV. The ion source temperature and interface temperature were both set at 250 °C with a solvent delay of 4 min. The quadrupole mass detector was set to 230 °C. The mass scanning range was *m*/*z* 30–600. A vacuum system equipped with a molecular turbine pump was utilized.

#### 2.6.3. Identification and Quantification of Volatile Compounds

The GC-MS data were retrieved and compounds were identified by searching against the NIST 17.LIB and NIST 17s.LIB standard spectral libraries. Compounds with a similarity higher than 80% were retained. The calculate retention indices (RI-cal) of the compounds were calculated using a mixture of n-alkanes and compared qualitatively with the literature RI (RI-lit) (https://webbook.nist.gov/chemistry/, accessed on 16 December 2023). The quantitative method utilized ethyl ester as an internal standard. The RI-cal were calculated as follows:

(1) $$\mathrm{RI}=100n+100\times \frac{RT_{x}-RT_{n}}{RT_{n+1}-RT_{n}}$$

where *T_x_* is the retention time of the compound to be measured; *n* is the number of carbon atoms in the n-alkane mixture; and *T_n_* and *T_n_*_+1_ are the retention times of the n-alkane standard mixture with n and n + 1 carbon atoms, respectively.

The determination of odor activity value (OAV) involved dividing the concentration of a volatile compound by its odor threshold values in water [16]. Principally, volatile compounds with OAV > 1 were deemed significant in terms of contributing to the overall aroma of the samples being analyzed.

### 2.7. Determination of Main Non-Volatile Compounds

#### 2.7.1. Determination of Water Extract, Tea Polyphenols, and Soluble Sugars

The water extract of Tuo tea was determined with the National Standard of China (GB/T 8305-2013) [17]. The tea polyphenols content of Tuo tea was determined by the Folin–Ciocalteu spectrophotometry method (GB/T 8313-2018) [18]. The soluble sugars content of Tuo tea was determined by the anthrone–sulfuric acid spectrophotometry method [19].

#### 2.7.2. HPLC Analysis of CAF, Catechins, GA, Flavones, and Flavonol Glycosides Content

The determination of CAF, catechins, GA, flavones, and flavonol glycosides involved using the Ultimate 3000 HPLC system (Thermo Fisher, Waltham, MA, USA) equipped with an Agilent Eclipse XDB-C18 column (4.6 mm × 250 mm, 5 μm). The flow rate of the mobile phase was 1 mL/min, and the sample injection volume was 10 μL. CAF, catechins, and GA content were simultaneously determined with the mobile phase consisting of a linear gradient elution of 0.2% acetic acid solution (A) and acetonitrile (B) under the following conditions: 0–12 min, 10% B to 13% B; 12–16 min, 13% B to 17% B; 16–22 min, 17% B; 22–25 min, 17% B to 32% B; 25–32 min, 32% B; 32–33 min, 32% B to 10% B; 33–40 min, 10% B. Chromatographic analysis was performed at a column temperature of 35 °C and detection at a wavelength of 278 nm using a UV detector.

The mobile phase consisted of 0.1% acetic acid solution (A) and acetonitrile (B) which were used to analyze flavones and flavonol glycosides content. The elution procedure was as follows: 0–5 min, 3% B to 16.5% B; 5–18 min, 16.5% B to 20% B; 18–23 min, 20% B to 21.2% B; 23–28 min, 21.2% B; 28–38 min, 21.2% B to 30% B; 38–48 min, 30% B; 48–50 min 30% B to 3% B; 50–60 min, 3% B. Absorbance at 360 nm was measured using a UV detector. The chromatographic determination was performed at the column temperature of 35 °C. The identified and quantified were based on the peak time and areas of the reference standards [20].

#### 2.7.3. HPLC Analysis of Free Amino Acids Content

The determination of free amino acids was carried out with slight modifications based on the Wan et al. method [21]. Free amino acids were analyzed using an HPLC with an Agilent EXTEND C18 column (4.6 mm × 250 mm, 5 μm) with an injection volume of 10 μL and detection wavelength of 360 nm using a UV detector. The mobile phase consisted of sodium acetate and tetrahydrofuran (4 mmol/L, pH = 5.7) (A) and acetonitrile/water (8:2, *v*/*v*) (B), and was eluted at a flow rate of 0.8 mL/min using a linear gradient according to the following conditions: 0–10 min, 5% B; 10–13 min, 5% B to 15% B; 13–16 min, 15% B to 16.8% B; 16–24 min, 16.8% B; 24–30 min, 16.8% B to 30% B; 30–50 min, 30% B to 65% B; 50–55 min, 65% B to 5% B; 52–60 min, 5% B at a column temperature of 35 °C. Each amino acid was identified and quantified based on the peak time and area of the reference standards.

### 2.8. Statistical Analysis

The statistical significance of the observed differences was assessed using Duncan’s multiple range tests and one-way ANOVA performed with SPSS version 25.0 (SPSS Inc., Chicago, IL, USA). PLS-DA was conducted by using SIMCA 14.1 software (Umetrics, Umea, Sweden). Heatmap visualization was performed using TBtools (JRE version 1.6, Guangzhou, China). Venn plot was drawn using Omicstudio (https://www.omicstudio.cn/, accessed on 16 December 2023). Graphs and charts were created using Origin 2023b software (Origin lab Inc., Northampton, MA, USA). All experiments were carried out in triplicate and all data were present by mean value ± SD.

## 3. Results and Discussion

### 3.1. Artificial Sensory Evaluation Analysis

Expert sensory evaluation is currently the most direct method to determine the quality grades, flavor characteristics, and sample differences [1]. As shown in Table 2, SG had the highest comprehensive score (93.17 ± 0.50), significantly outperforming the other samples (*p* < 0.05), whereas 2G had the lowest comprehensive score of 86.37 ± 0.42. All samples scored above 86 points. Specifically, in terms of aroma, SG exhibited a strong and long-lasting aged aroma, but this unique aroma gradually weakened in perception as the grade declined (*p* < 0.05). The taste of the SG was heavy and thick, and a noticeable aged aroma could be felt in the tissues of the mouth and nose while drinking tea infusion. The taste of the 1G was heavy and mellow, although not as heavy as that of the SG, while the 2G had a more noticeable astringency compared to the other two samples. The results demonstrated that sensory quality varies among different grades of Tuo teas even when subjected to identical processing and storage conditions. Higher-grade Tuo tea offers a more enjoyable sensory experience and receives higher ratings. This finding aligned with previous research, highlighting a strong correlation between tea quality and its grade [22].

### 3.2. Intelligent Sensory Evaluation Analysis

**Figure 1:**
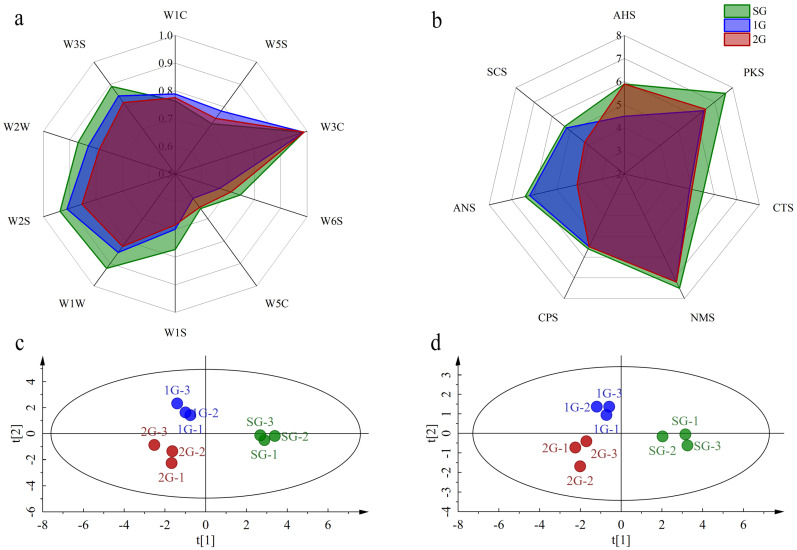
Electronic nose and electronic tongue analysis in the different grades of Tuo teas. Radar chart of electronic nose (**a**) and electronic tongue (**b**) sensor responses. PLS-DA score plots of electronic nose (**c**) and electronic tongue (**d**).

Although discernible distinctions in the quality of different grades of Tuo tea were observed through artificial sensory evaluation, there may be some biases in evaluating samples due to the subjectivity of this method [23]. The applications of rapid food inspection technology for the classification and grading of tea provide us with new insight [24,25]. In this study, we employed electronic nose and electronic tongue techniques to enhance the assessment of aroma and taste in Tuo tea. The result of the electronic nose was illustrated in Figure 1a. Remarkable variations in the sensor response values of different samples were acquired, except for W3C. Specifically, SG had lower response values on the W1C and W5S sensors compared to other samples. As shown in Figure 1b, the radar chart of taste intensity for different grades of Tuo teas using electronic tongue exhibited different taste profiles, primarily manifested in the ANS, AHS, SCS, and PKS sensors. SG had the strongest intensity in each taste attribute compared to other samples, while 1G was significantly lower than other samples in the AHS. The intensity of 2G was significantly lower than other samples in ANS, SCS, and PKS, while the stronger astringency expressed by 2G was not reflected in the radar chart, possibly due to the sensory evaluation being a comprehensive result of overall the taste, whereas the results of the electronic tongue were limited by the types of sensors used.

PLS-DA score plot of the electronic nose showed that samples from three distinct grades were well separated without overlapping, declaring differences in the aroma profile of Tuo tea of varying grades (R^2^Y = 0.983 and Q^2^ = 0.941) (Figure 1c). These findings demonstrated that the electronic nose analysis can effectively differentiate different grades of Tuo teas and had good reproducibility, which was consistent with previous studies on jasmine tea [14]. The PLS-DA result of the electronic tongue (R^2^Y = 0.978 and Q^2^ = 0.963) was similar to that of the electronic nose, with different grades of Tuo tea dispersed in different regions (Figure 1d). These findings provided evidence that the electronic tongue was capable of effectively distinguishing between samples and emphasizing the taste variations present among them. In conclusion, variations in both aroma and taste existed among different grades of Tuo tea, aligning with sensory outcomes and affirming the potential for utilizing electronic nose and electronic tongue to swiftly differentiate between various grade samples based on the distinctions in aroma and the taste of Tuo tea.

### 3.3. Analysis of Volatile Compounds

#### 3.3.1. Volatile Compounds as Analyzed via GC-MS

**Figure 2:**
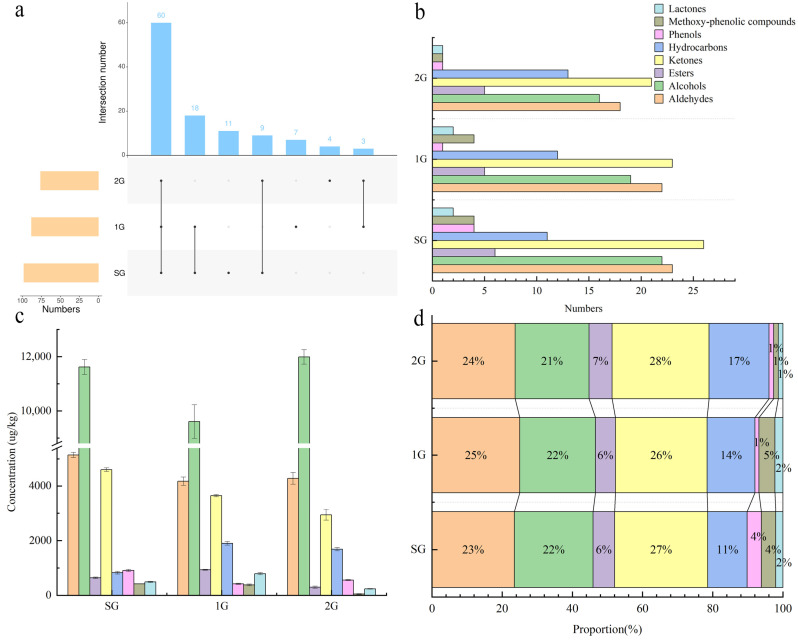
The Venn plot (**a**) and histogram of numbers (**b**), concentration (**c**), and proportion (**d**) of each volatile compound content category of total volatile compounds in the different grades of Tuo teas.

Aroma, as one of the important factors affecting tea consumption, is a comprehensive reflection of the different ratios and thresholds of aromatic compounds contained in tea [26]. The results of GC-MS for different grades of Tuo tea were shown in Table 3. A total of 112 volatile compounds were detected in the three samples, classified into seven categories, including 29 ketones, 26 aldehydes, 22 alcohols, 17 hydrocarbons, 8 esters, 4 phenolic compounds, 4 methoxy–phenolic compounds, and 2 lactones. A total of 60 compounds common to all three samples were detected, out of which 56 exhibited significant differences in content (*p* < 0.05). The Venn plot depicted that SG, 1G, and 2G contained 98, 88, and 76 compounds, respectively. The total number of compounds decreased with the reduction in grades and the number of volatile compounds that Tuo tea shares or that are unique (Figure 2a). In SG, a total of 11 unique volatile compounds were identified, namely 3,5-dimethylphenol, 4-ethylphenol, benzyl acetate, 4-ethyl-2-methoxyphenol, n-dodecanal, caprylic acid methyl ester, 2-nonanone, 2-heptanone, benzyl alcohol, epiglobulol, 6,10-dimethyl-2-undecanone. Conversely, (2E,6E)-3,7,11-trimethyldodeca-2,6,10-trienal, (Z)-calamenene, 1-(2-pyrrolyl)-1-ethanone, β-cedrene, isopropyl salicylate, dehydro-ar-ionene and dihydro-β-ionone were exclusively detected in 1G, while four volatile compounds, β-myrcene, citral, (E)-2-decenal and ocimene were found solely in 2G.

The number of ketone compounds was the highest among all the teas (Figure 2b). But ketone compounds content was lower than that of alcohol compounds, the higher content of alcohol compounds, such as linalool and its oxide, 4-terpineol, and (R)-(+)-α-terpineol, in the samples is primarily responsible for this phenomenon (Figure 2c). Ketones (26.14~27.63%) were the predominant volatile compounds in the tea samples, followed by aldehydes (23.47~25.00%), alcohols (21.05~22.45%), and hydrocarbons (11.22~17.11%). Collectively, these compounds accounted for over 80% of the total volatile compounds (Figure 2d). This is consistent with the results of the electronic nose. The total content of volatile compounds in Tuo tea of various grades, ranking from highest to lowest, was observed as SG (24.66 mg/kg), 2G (22.03 mg/kg), and 1G (21.85 mg/kg), but there was no significant difference between 1G and 2G. Furthermore, some volatile compounds exhibited consistent variations in their content relative to the grade of tea. For instance, 6-methyl-5-hepten-2-one, β-cyclocitral, 4-ethyl-1,2-dimethoxybenzene, β-damascenone and α-ionone levels decreased as the grade decreased, whereas octanal, (S)-(−)-limonene, (E)-3, 7-dimethylocta-1,3,6-triene, 4-terpineol, (R)-(+)-α-terpineol and geraniol demonstrated the opposite trend.

#### 3.3.2. OAV Analysis of Key Odor-Active Compounds

The OAV is widely employed to identify key odor-active compounds in tea, with OAV > 1 being generally regarded as playing a significant role in determining characteristic aromas [27]. A higher OAV indicates a stronger contribution of the compound to aroma characteristics [24]. Table 4 revealed variations and quantities of volatile compounds with OAV > 1 across different grades of Tuo teas. A total of 56 volatile compounds with OAV > 1 were detected in all three grades of Tuo tea. Among SG, 1G, and 2G, there were 46, 43, and 42 of volatile compounds with OAV > 1, respectively. Collectively, all three grades identified a total of 32 volatile compounds with OAV > 1, comprising 9 aldehydes, 9 ketones, 8 alcohols, and 5 hydrocarbons. In SG, the five compounds with the highest OAV were identified as (E,E)-alloocimene, safranal, linalool, (E,Z)-2,4-decadienal, and geraniol. These compounds are primarily responsible for imparting floral, sweet fruity, and woody aromas [16,27]. (E,Z)-2,4-decadienal, (E,E)-alloocimene, cedrol, linalool, and (E)-2-nonenal exhibit relatively higher OAV in 1G. Among 2G, the top five compounds with the highest OAV were identified as (Z)-jasmone, geraniol, linalool, safranal, and (E)-2-nonenal. However, among these compounds, except for linalool, which showed consistency with the previous two grades, the other key odor-active compounds exhibited significant variations. Linalool and cedrol have been identified as the key volatile contributors to the arohid and aged aroma of Fu Brick tea [28].

Nevertheless, it is noteworthy that previous studies have regarded methoxy–phenolic compounds as significant contributors to the aged aroma of tea [29]. Nonetheless, the extent to which methoxy–phenolic compounds contribute to the aged aroma of Tuo tea requires further exploration, primarily due to the absence of relevant thresholds for three out of the four detected methoxy–phenolic compounds (1,2-dimethoxybenzene, 1,2,3-trimethoxybenzene, and 4-ethyl-1,2-dimethoxybenzene) in Tuo tea.

**Table 4:** The thresholds, OAV, VIP and significance of the volatile compounds found in the different grades of Tuo teas.

No.	Compounds	Threshold (μg/kg) ^a^	OAV			VIP	Significance
			SG	1G	2G		
#1	Hexanal	5.00	4.24	4.60	6.64	1.0181	0.000
#2	(E)-2-Hexenal	88.50	0.28	0.24	n.f.	0.9234	0.013
#3	2-Heptanone	140.00	0.29	n.f.	n.f.	1.0075	0.000
#4	1-Heptanal	2.80	50.34	46.75	n.f.	1.0240	0.000
#5	(E)-2-Heptenal	51.00	1.64	0.70	4.82	1.0556	0.000
#6	Benzaldehyde	350.00	1.90	1.03	1.28	0.9479	0.002
#7	3,5,5-Trimethyl-hex-1-ene	n.f. ^b^	n.f.	n.f.	n.f.	1.0205	0.001
#8	1-Octen-3-one	4.00	17.75	8.23	35.23	1.0374	0.000
#9	1-Octen-3-ol	1.50	198.23	102.27	313.19	1.0538	0.000
#10	6-Methyl-5-hepten-2-one	68.00	5.09	2.70	1.27	1.0310	0.000
#11	β-Myrcene	1.20	n.f.	n.f.	312.94	1.0590	0.000
#12	(E,Z)-2,4-Heptadienal	94.80	7.76	5.88	6.08	1.0041	0.000
#13	Octanal	0.59	220.15	251.11	301.46	0.9845	0.001
#14	(E,E)-2,4-Heptadienal	15.40	54.80	49.04	49.41	0.9341	0.004
#15	p-Cymene	5.01	7.58	19.40	13.07	1.0067	0.000
#16	(S)-(−)-limonene	1040.00	0.15	0.24	0.32	0.9789	0.001
#17	3-Octen-2-one	250.00	0.13	0.02	0.12	0.9891	0.001
#18	Ocimene	34.00	n.f.	n.f.	4.12	1.0603	0.000
#19	(2E,6E)-3,7,11-trimethyldodeca-2,6,10-trienal	n.f.	n.f.	n.f.	n.f.	1.0412	0.000
#20	Benzyl alcohol	2546.21	0.07	n.f.	n.f.	1.0186	0.000
#21	(E)-3,7-Dimethylocta-1,3,6-triene	34.00	0.83	2.00	2.50	1.0276	0.000
#22	Phenylacetaldehyde	6.30	19.27	15.79	25.57	0.9869	0.003
#23	1-Ethyl-1H-pyrrole-2-carbaldehyde	65,000.00	0.01	0.00	0.00	1.0454	0.000
#24	(E)-2-Decenol	n.f.	n.f.	n.f.	n.f.	1.0605	0.000
#25	(E)-2-Octenal	3.00	84.27	58.51	94.61	1.0294	0.000
#26	Acetophenone	65.00	2.73	n.f.	2.29	1.0308	0.000
#27	1-(2-Pyrrolyl)-1-ethanone	58,585.25	n.f.	0.00	n.f.	1.0380	0.000
#28	(E)-Linalool oxide (Furan type)	190.00	6.47	3.20	3.40	1.0117	0.000
#29	1-Octanol	23.00	8.15	3.24	5.47	1.0030	0.000
#30	2-Nonyn-1-ol	n.f.	n.f.	n.f.	n.f.	0.9962	0.000
#31	Terpinolene	200.00	0.65	0.69	0.45	0.9680	0.005
#32	(Z)-Linalool oxide (Furan type)	100.00	12.48	4.53	4.66	1.0158	0.000
#33	2-Nonanone	200.00	0.25	n.f.	n.f.	1.0053	0.000
#34	(E,E)-3,5-Octadien-2-one	100.00	4.19	1.32	1.24	1.0156	0.000
#35	Linalool	6.00	480.09	469.41	541.84	0.8862	0.028
#36	3,7-Dimethylocta-1,5,7-trien-3-ol	110.00	8.54	2.90	9.76	1.0483	0.000
#37	Nonanal	3.10	157.85	137.21	148.74	0.7415	0.107
#38	Isophorone	1.70	13.89	22.46	24.80	0.8868	0.016
#39	Caprylic acid methyl ester	200.00	0.09	n.f.	n.f.	1.0126	0.000
#40	(E,E)-Alloocimene	0.03	2083.00	1237.82	n.f.	1.0316	0.000
#41	(E)-3-Nonen-2-one	n.f.	n.f.	n.f.	n.f.	0.9267	0.013
#42	2,6,6-Trimethyl-2-cyclohexene-1,4-dione	n.f.	n.f.	n.f.	n.f.	0.9898	0.000
#43	1,2-Dimethoxybenzene	n.f.	n.f.	n.f.	n.f.	1.0577	0.000
#44	3,5-Dimethylphenol	5000.00	0.01	n.f.	n.f.	1.0185	0.000
#45	4-(5-Methyl-2-furyl)butan-2-one	n.f.	n.f.	n.f.	n.f.	1.0427	0.000
#46	(2E,6Z)-2,6-Dodecadienal	n.f.	n.f.	n.f.	n.f.	1.0309	0.000
#47	(E)-2-Nonenal	0.19	371.43	458.15	327.34	0.7326	0.140
#48	Benzyl acetate	364.00	0.16	n.f.	n.f.	1.0172	0.000
#49	4-Ethylphenol	13.00	5.88	n.f.	n.f.	1.0189	0.000
#50	(E)-Linalool oxide (Pyran type)	500.00	0.50	0.15	0.25	1.0074	0.000
#51	(Z)-Linalool oxide (Pyran type)	500.00	1.49	0.60	0.74	1.0056	0.000
#52	4-Terpineol	1200.00	0.38	0.54	0.64	1.0092	0.000
#53	4-Methylacetophenone	21.00	3.70	2.26	2.11	0.9698	0.001
#54	p-Cymen-8-ol	n.f.	n.f.	n.f.	n.f.	1.0416	0.000
#55	Methyl salicylate	40.00	11.08	1.97	3.84	1.0121	0.000
#56	(R)-(+)-α-Terpineol	6800.00	0.29	0.45	0.53	1.0099	0.000
#57	Safranal	0.70	569.34	371.20	437.26	0.9471	0.002
#58	Decyl aldehyde	3.00	77.97	95.40	132.85	1.0221	0.000
#59	3,5-Dimethylbenzaldehyde	n.f.	n.f.	n.f.	n.f.	1.0597	0.000
#60	β-Cyclocitral	5.00	62.02	32.06	4.46	1.0319	0.000
#61	Nerol	680.00	0.31	0.28	0.29	0.4139	0.581
#62	3,4-Dimethoxytoluene	1.44	115.91	50.44	n.f.	1.0286	0.000
#63	(E)-Thujone	n.f.	n.f.	n.f.	n.f.	1.0123	0.000
#64	Geraniol	1.10	417.24	160.45	552.62	1.0426	0.000
#65	(E)-2-Decenal	17.00	n.f.	n.f.	8.67	1.0426	0.000
#66	Citral	40.00	n.f.	n.f.	1.52	1.0288	0.000
#67	4-Ethyl-2-methoxyphenol	89.25	3.33	n.f.	n.f.	1.0193	0.000
#68	1-Methylnaphthalene	7.50	5.37	n.f.	7.28	1.0352	0.000
#69	2-Undecanone	5.50	8.50	9.22	n.f.	1.0543	0.000
#70	(E,Z)-2,4-Decadienal	0.04	431.94	1680.14	n.f.	1.0518	0.000
#71	Isopropyl salicylate	n.f.	n.f.	n.f.	n.f.	1.0413	0.000
#72	1,2,3-Trimethoxybenzene	n.f.	n.f.	n.f.	n.f.	1.0578	0.000
#73	Theaspirane	1000.00	0.08	0.18	0.12	0.9726	0.001
#74	4-Ethyl-1,2-dimethoxybenzene	n.f.	n.f.	n.f.	n.f.	0.9890	0.001
#75	3-Nonen-2-one	800.00	0.06	0.03	n.f.	1.0368	0.000
#76	Dehydro-ar-ionene	2.50	n.f.	46.82	n.f.	1.0334	0.000
#77	γ-Nonalactone	9.70	4.75	5.18	n.f.	0.9959	0.002
#78	2-Undecenal	n.f.	n.f.	n.f.	n.f.	1.0009	0.000
#79	β-Damascenone	10.00	17.82	11.54	2.52	1.0360	0.000
#80	(Z)-Jasmone	0.26	314.59	263.02	668.54	1.0208	0.000
#81	6,10-Dimethyl-2-undecanone	n.f.	n.f.	n.f.	n.f.	0.9993	0.000
#82	Dodecanal	33.00	1.02	n.f.	n.f.	1.0189	0.000
#83	Dihydro-α-ionone	n.f.	n.f.	n.f.	n.f.	1.0588	0.000
#84	α-Ionone	3.78	152.58	113.06	67.80	1.0206	0.000
#85	Dihydro-β-ionone	1.00	n.f.	175.77	n.f.	1.0354	0.000
#86	4-(2,2-Dimethyl-6-methylenecyclohexyl)butan-2-one	n.f.	n.f.	n.f.	n.f.	1.0094	0.000
#87	6,10-Dimethyl-5,9-undecadien-2-one	60.00	8.00	4.34	4.55	1.0141	0.000
#88	Caryophyllene	64.00	2.71	0.63	3.61	1.0331	0.000
#89	2,6-Di(tert-butyl)-4-hydroxy-4-methyl-2,5-cyclohexadien-1-one	n.f.	n.f.	n.f.	n.f.	0.9827	0.001
#90	(−)-Alloaromadendrenepurum	n.f.	n.f.	n.f.	n.f.	0.9982	0.000
#91	4-Tert-butyl phenylacetone	n.f.	n.f.	n.f.	n.f.	0.9832	0.003
#92	β-Ionone	8.40	92.05	72.07	82.13	0.9800	0.000
#93	5,6-Epoxy-β-ionone	n.f.	n.f.	n.f.	n.f.	0.8974	0.011
#94	β-Cedrene	n.f.	n.f.	n.f.	n.f.	1.0418	0.000
#95	1,5-Cyclodecadiene,1,5-dimethyl	n.f.	n.f.	n.f.	n.f.	1.0052	0.000
#96	2,4-Ditert-butylphenol	500.00	0.99	0.84	1.11	0.9586	0.006
#97	(Z)-Calamenene	n.f.	n.f.	n.f.	n.f.	1.0355	0.000
#98	Dihydroactinidiolide	500.00	0.89	1.48	0.47	1.0526	0.000
#99	Nerolidol	250.00	0.29	0.10	n.f.	1.0023	0.000
#100	Spathulenol	n.f.	n.f.	n.f.	n.f.	1.0443	0.000
#101	2,2,4-Trimethylpentanediol-1,3-diisobutyrate	n.f.	n.f.	n.f.	n.f.	0.5174	0.407
#102	Caryophyllene oxide	200.00	0.16	n.f.	0.15	0.9936	0.001
#103	Cedrol	0.50	88.21	545.63	275.98	1.0200	0.000
#104	Tridecane aldehyde	70.00	0.36	1.09	n.f.	1.0404	0.000
#105	Epiglobulol	n.f.	n.f.	n.f.	n.f.	1.0059	0.000
#106	α-Cadinol	n.f.	n.f.	n.f.	n.f.	0.9882	0.002
#107	Isopropyl myristate	n.f.	n.f.	n.f.	n.f.	0.7333	0.142
#108	Hexahydrofarnesyl acetone	n.f.	n.f.	n.f.	n.f.	1.0328	0.000
#109	Diisobutyl phthalate	n.f.	n.f.	n.f.	n.f.	1.0318	0.000
#110	Dibutyl phthalate	n.f.	n.f.	n.f.	n.f.	1.0100	0.000
#111	(Z)-7-Hexadecenal	n.f.	n.f.	n.f.	n.f.	1.0425	0.000
#112	Phytol	640.00	0.01	0.09	0.01	1.0401	0.000

Note: ^a^ The threshold of volatile compounds in water referred to references [30,31,32,33] and literature [34]. Compilations of odor threshold values in air, water, and other media; ^b^ n.f.: threshold or OAV was not found.

#### 3.3.3. Multivariate Statistical Analysis

**Figure 3:**
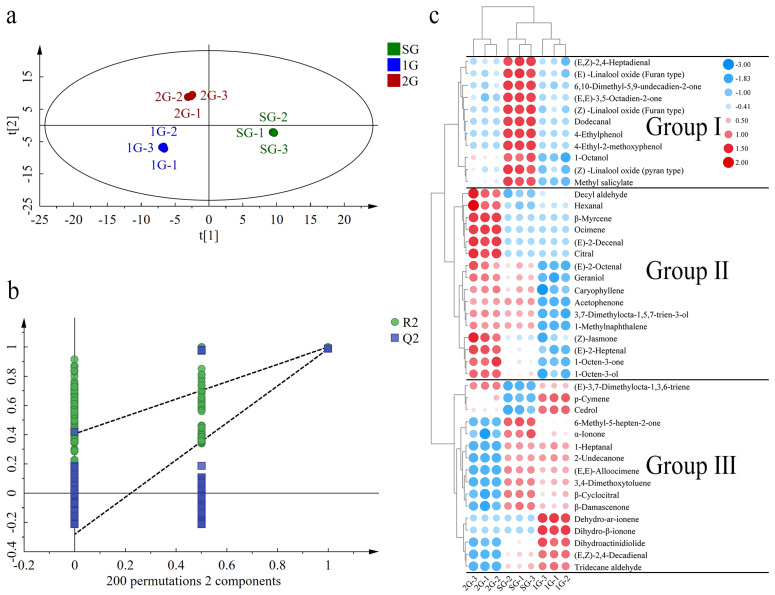
Multivariate statistical analysis of volatile compounds in different grades of Tuo teas via GC-MS. PLS-DA score plot (**a**). Cross-validation results with 200 times permutation test (**b**). Heatmap visualization (**c**).

To further investigate the relationship between volatile compounds and three grades of Tuo tea, PLS-DA was conducted on a total of 112 volatile compounds. The results of the PLS-DA model revealed that SG clusters in the fourth quadrant, 2G clusters in the second quadrant, and 1G clusters in the third quadrant (Figure 3a). Consequently, the three grades of Tuo tea can be segregated into three distinct groups. The model’s fitting parameters demonstrated high accuracy, with R^2^X = 0.926, R^2^Y = 0.999, and Q^2^ = 0.996, indicating significant differences in volatile compounds among the three grades of Tuo tea. To ensure the reliability of the model, permutation tests were conducted through 200 times cross-validations (Figure 3b). The intercept of the Q^2^ regression line with the y-axis was less than zero, suggesting that the PLS-DA model was not overfitting and could be considered reliable (R^2^ = 0.347, Q^2^ = −0.310).

In PLS-DA discrimination, it is common to adopt a VIP > 1 in conjunction with *p* < 0.05 as the criteria for selecting discriminatory compounds [35]. All VIP and *p*-values were listed in Table 3. Out of the different grades of Tuo teas, a total of 80 compounds with VIP > 1 and *p* < 0.05 were identified. By further screening with OAV > 1, 43 compounds were ultimately selected for distinguishing between the various grades of Tuo teas. For heatmap and clustering analysis, 43 compounds were categorized into three groups (Figure 3c). Among them, 11 compounds were recognized as key differential volatile compounds in SG. These primarily included linalool oxides with a floral and sweet woody aroma, floral compounds such as (E,Z)-2,4-heptadienal, 6,10-dimethyl-5,9-undecadien-2-one, and dodecanal, as well as wintergreen mint-like methyl salicylate and spicy aroma 4-ethylphenol, 4-ethyl-2-methoxyphenol, and 1-octanol [36,37]. Notably, linalool and its oxides were reported as key aroma compounds in high-quality tea leaves [8]. In 1G, a total of 16 volatile compounds were identified as key differential volatile compounds. These included (E)-3,7-dimethylocta-1,3,6-triene, 1-heptanal, and dehydro-ar-ionene with an herbal aroma, as well as 3,4-dimethoxytoluene with an aged aroma and cedarwood woody dry sweet soft cedrol. Cedrol, derived mainly from glycoside hydrolysis, was found to be the primary contributing compound to the aroma of dark tea [27,29]. Similarly, 16 volatile compounds were determined to be key differential volatile compounds in 2G. This group included 3,7-dimethylocta-1,5,7-trien-3-ol with a moldy and woody aroma, and (E)-2-decenal, 1-octen-3-one, and 1-octen-3-ol with mushroom aroma. These compounds might explain the sensory presentation of arohid fragrance in 2G [27,37]. These compounds play crucial roles in distinguishing the aroma characteristics of Tuo tea across different grades. However, it is worth noting that certain compounds such as 3,5-dimethylbenzaldehyde, and (E)-2-decenol possess a VIP > 1 and *p* < 0.05 but lack a defined threshold, rendering the analysis mentioned above inconclusive.

### 3.4. Analysis of Main Non-Volatile Compounds

#### 3.4.1. Content Analysis of Main Non-Volatile Compounds

The water extract, CAF, GA, tea polyphenols, soluble sugars, 11 types of flavones and flavonol glycosides, 20 types of free amino acids, and 8 types of catechins were analyzed in Tuo tea of three grades. Table 5 indicated differences in the water extract content of Tuo tea. Water extract is thought to have a positive correlation with taste quality, implying that the decrease in Tuo tea grade may be linked to reduced taste component quantities [38]. This is similar to the research results of Su et al. [4]. No significant differences were found in the contents of tea polyphenols, soluble sugars, CAF, and total catechins among the three grades of Tuo tea. In contrast, there were variations in the content of different catechins. The content of ester catechins varied across the three grades of Tuo tea, and these differences might have contributed to SG and 1G, which exhibited more mellow results than 2G. The total flavones and flavonol glycosides declined as the Tuo tea grade decreased, while it suggested the inverse trend for the total content of free amino acids. Flavones and flavonol glycosides in 2G were considerably more abundant than in other samples (excluding kaempferol 3-O-rutinoside (Kae-rut) and myricetin (Myr)) (*p* < 0.05). These compounds are often responsible for the astringent flavor of tea, such as kaempferol 3-O-glucopyranoside (Kae-gluc) have significantly lower astringent taste thresholds than bitterness thresholds [39,40]. The higher levels of flavones and flavonol glycosides in 2G might contribute to its more pronounced astringency compared to SG and 1G. The quality of tea is significantly affected by the composition, content, and related derivatives of free amino acids [21]. Various free amino acids’ contents differed significantly across the tea samples (excluding Alanine (Ala), Aspartic acid (Asp), and Lysine (Lys)), with Thea exhibiting the highest amino acid content in Tuo tea. The content order of GA was 1G > 2G > SG, and its correlation with tea grade requires further investigation.

#### 3.4.2. Multivariate Statistical Analysis

**Figure 4:**
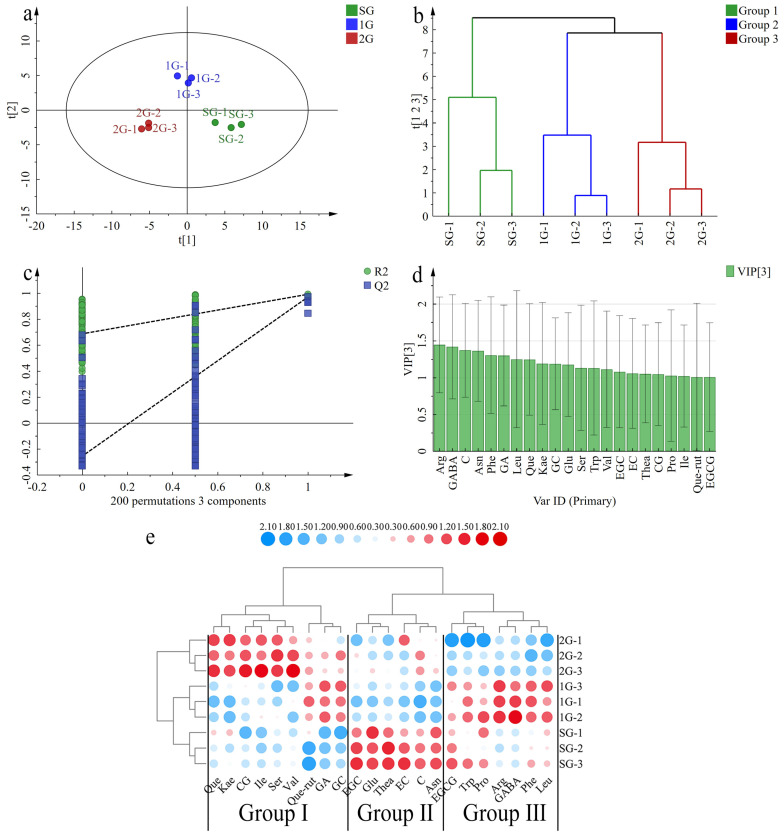
Multivariate statistical analysis of non-volatile compounds in different grades of Tuo teas. PLS-DA score plot (**a**). HCA (**b**). Cross-validation results with 200 times permutation test (**c**). VIP > 1 plot (**d**). Heatmap visualization (**e**).

To identify the key differential non-volatile compounds among different grades of Tuo teas, PLS-DA analysis was conducted on 44 non-volatile compounds. The fitting indexes of the PLS-DA model were as follows: R^2^X for independent variables was 0.825, R^2^Y for dependent variables was 0.992, and Q^2^ for model prediction was 0.972 (Figure 4a). Figure 4b showed that HCA divided the three grades of Tuo tea into three categories, allowing SG, 1G, and 2G to be differentiated according to non-volatile compounds content. Figure 4c depicted the validation of the PLS-DA rationale by performing 200 times permutations using a cross-validation model, yielding R^2^ = 0.698 and Q^2^ = −0.156. Figure 4d displayed the results of VIP > 1, which identified 22 compounds as differential markers for the three grades of Tuo tea. These included GA, 3 flavones and flavonol glycosides, 6 catechins, and 12 free amino acids. The heatmap and clustering analysis demonstrated that the 22 compounds could be grouped into three categories (Figure 4e). Six of these compounds were identified as key differential non-volatile compounds in SG, such as the umami amino acids (Thea and Asn), as well as the bitter-tasting (Glu, EC, EGC, and C) compounds [41,42]. A total of 7 non-volatile compounds, including EGCG, Trp, Pro, Arg, GABA, Phe, and Leu, were identified as key differential non-volatile compounds in 1G. These compounds were primarily composed of sweet and umami-tasting free amino acids [42]. Nine non-volatile compounds, including GA, GC, CG, Que, Kac, Que-rut, Ile, Ser, and Val, were identified as key differential non-volatile compounds in 2G. Flavones and flavonol glycosides can intensify the bitterness of tea infusion by enhancing CAF’s bitterness [43]. The less coordinated taste of 2G when compared to SG and 1G might be due to the presence of Que, Kac, and Que-rut. These findings suggest that the variations in the material basis of Tuo tea contribute to the differences in taste among different grades.

## 4. Conclusions

Tuo tea of various grades possesses unique flavor characteristics. The findings from sensory evaluation, electronic tongue, and electronic nose suggested that Tuo tea of various grades showed differences in both aroma and taste. A total of 112 volatile compounds were identified through GC-MS, out of which 56 exhibited OAV > 1 in the three grades of Tuo tea. Variances were observed in the content of 44 non-volatile compounds among the three grades of Tuo tea. PLS-DA analysis was conducted on 112 volatile compounds and 44 non-volatile compounds, resulting in satisfactory model parameters. Among the volatile compounds, a total of 80 key differential compounds were identified (VIP > 1, *p* < 0.05); 43 compounds possessed OAV > 1 and were identified as the odor-active compounds that differentiate the three grades of Tuo tea. From the non-volatile compounds, 22 key differential non-volatile compounds were selected to distinguish the three grades of Tuo tea. Hence, the variations in composition and compound content are responsible for the differences in quality among Tuo tea grades. Nevertheless, additional research is necessary to investigate the flavor mechanisms and formation processes of key differential compounds. To the best of our knowledge, this study represented the initial comprehensive characterization of compounds in Tuo tea of various grades using techniques like electronic nose, electronic tongue, and GC-MS. These novel findings provided a theoretical basis for future investigations and propose a new method to distinguish Tuo tea grades.

## Figures and Tables

**Table 1:** Sensitive substances of electronic nose sensor.

No.	Sensor	Response Substance
S1	W1C	Aromatic compounds
S2	W5S	Nitrogen oxides
S3	W3C	Ammonia, aromatic compounds
S4	W6S	Hydrocarbons, aromatic compounds
S5	W5C	Short-chain alkanes
S6	W1S	Alkanes, methyl compounds
S7	W1W	Pyrazine, terpenes, inorganic sulfides, etc.
S8	W2S	Alcohols, aldehydes, ketones
S9	W2W	Aromatic, organic sulfides
S10	W3S	Long-chain alkanes

**Table 2:** Sensory evaluation in the different grades of Tuo teas.

Sample ID	Appearance	Liquor Color	Aroma	Taste	Infused Leaves	Total Score ^a^
SG	green and bloom, slightly tippy	bright orange	strong and lasting aged aroma	heavy and thick	soft and bright	93.17 ± 0.50 a
1G	green and bloom, fairly tippy	still bright orange	comparatively aged aroma	heavy and mellow	still soft and bright	89.93 ± 0.55 b
2G	still green and bloom	still bright orange	slightly aged with an arohid aroma	mellow-thick with slightly astringent	uneven, still soft, and bright	86.37 ± 0.42 c

Note: ^a^ Different lowercase letters marked on the different row represent statistically significant differences in the data (*p* < 0.05).

**Table 3:** The content of identified volatile compounds in the different grades of Tuo teas via GC-MS.

No.	Compounds	CAS#	Rt ^a^	RI-cal	RI-lit	ID ^b^	Contents (μg/kg) ^c^		
							SG	1G	2G
#1	Hexanal	66-25-1	6.277	802	802	MS, RI	21.18 ± 0.92 a	23.01 ± 0.12 a	33.18 ± 2.95 b
#2	(E)-2-Hexenal	6728-26-3	8.559	851	855	MS, RI	25.22 ± 3.63 a	21.6 ± 12.62 b	n.d. ^d^
#3	2-Heptanone	110-43-0	10.295	889	889	MS, RI	40.17 ± 6.19	n.d.	n.d.
#4	1-Heptanal	111-71-7	11.052	904	896	MS, RI	140.96 ± 12.12 a	130.91 ± 33.74 a	n.d.
#5	(E)-2-Heptenal	18829-55-5	14.15	953	978	MS, RI	83.71 ± 10.03 b	35.81 ± 8.38 c	245.80 ± 20.49 a
#6	Benzaldehyde	100-52-7	14.315	956	961	MS, RI	664.75 ± 44.97 a	359.48 ± 19.47 b	446.58 ± 93.63 b
#7	3,5,5-Trimethyl-hex-1-ene	4316-65-8	15.242	970	n.f. ^e^	MS	43.24 ± 3.09 b	28.62 ± 5.68 c	69.68 ± 8.45 a
#8	1-Octen-3-one	4312-99-6	15.433	973	980	MS, RI	70.99 ± 9.16 b	32.94 ± 1.84 c	140.92 ± 19.53 a
#9	1-Octen-3-ol	3391-86-4	15.714	978	964	MS	297.34 ± 15.46 b	153.4 ± 31.90 c	469.79 ± 4.44 a
#10	6-Methyl-5-hepten-2-one	110-93-0	16.057	983	986	MS, RI	345.91 ± 14.24 a	183.86 ± 1.58 b	86.14 ± 0.26 c
#11	β-Myrcene	123-35-3	16.203	991	991	MS, RI	n.d.	n.d.	375.53 ± 21.03
#12	(E,Z)-2,4-Heptadienal	5910-85-0	16.723	994	999	MS, RI	735.44 ± 7.56 a	557.82 ± 25.52 b	576.55 ± 8.49 b
#13	Octanal	124-13-0	17.23	1002	1001	MS, RI	129.23 ± 5.56 c	147.40 ± 13.45 b	176.96 ± 3.58 a
#14	(E,E)-2,4-Heptadienal	4313/3/5	17.698	1008	1015	MS, RI	843.84 ± 21.20 a	755.28 ± 28.50 b	760.93 ± 11.28 b
#15	p-Cymene	99-87-6	18.463	1019	1024	MS, RI	37.98 ± 1.57 c	97.19 ± 2.64 a	65.50 ± 8.33 b
#16	(S)-(−)-limonene	5989-54-8	18.746	1023	1028	MS, RI	153.83 ± 6.49 c	249.75 ± 40.52 b	331.58 ± 35.25 a
#17	3-Octen-2-one	1669-44-9	19.57	1034	1040	MS, RI	31.33 ± 1.57 a	6.08 ± 1.25 b	29.93 ± 7.70 a
#18	Ocimene	13877-91-3	20.21	1043	1026	MS	n.d.	n.d.	140.11 ± 3.58
#19	(2E,6E)-3,7,11-trimethyldodeca-2,6,10-trienal	502-67-0	20.595	1048	1730	MS	n.d.	30.93 ± 1.16	n.d.
#20	Benzyl alcohol	100-51-6	19.267	1030	1034	MS, RI	189.20 ± 7.61	n.d.	n.d.
#21	(E)-3,7-Dimethylocta-1,3,6-triene	3779-61-1	19.45	1033	1052	MS	28.15 ± 0.95 c	67.9 ± 0.36 b	84.83 ± 2.61 a
#22	Phenylacetaldehyde	122-78-1	19.78	1037	1043	MS	121.4 ± 8.90 b	99.48 ± 7.95 b	161.07 ± 18.25 a
#23	1-Ethyl-1H-pyrrole-2-carbaldehyde	2167-14-8	20.03	1041	1046	MS, RI	370.70 ± 9.95 a	289.66 ± 21.06 b	97.91 ± 3.41 b
#24	(E)-2-Decenol	18409-18-2	20.881	1052	1251	MS	115.87 ± 5.23 a	122.26 ± 2.19 a	n.d.
#25	(E)-2-Octenal	2548-87-0	21.026	1054	1062	MS, RI	252.81 ± 8.67 b	175.53 ± 2.49 c	283.84 ± 19.40 a
#26	Acetophenone	98-86-2	21.25	1058	1078	MS, RI	177.36 ± 1.88 a	n.d.	148.77 ± 14.37 b
#27	1-(2-Pyrrolyl)-1-ethanone	1072-83-9	21.295	1058	1072	MS, RI	n.d.	224.05 ± 19.60	n.d.
#28	(E)-Linalool oxide (Furan type)	34995-77-2	21.739	1064	1070	MS, RI	1229.95 ± 32.54 a	608.11 ± 51.13 b	646.64 ± 32.84 b
#29	1-Octanol	111-87-5	21.961	1067	1078	MS, RI	187.40 ± 10.07 a	74.45 ± 7.01 c	125.92 ± 12.81 b
#30	2-Nonyn-1-ol	5921-73-3	22.267	1072	1105	MS	104.88 ± 3.78 a	64.55 ± 7.06 c	92.94 ± 4.57 b
#31	Terpinolene	586-62-9	22.728	1078	1088	MS, RI	129.92 ± 4.89 a	138.5 ± 19.85 a	89.02 ± 4.12 b
#32	(Z)-Linalool oxide (Furan type)	5989-33-3	22.906	1081	1080	MS, RI	1248.20 ± 22.86 a	453.30 ± 58.90 b	466.32 ± 8.65 b
#33	2-Nonanone	821-55-6	23.375	1087	1090	MS, RI	49.31 ± 8.30	n.d.	n.d.
#34	(E,E)-3,5-Octadien-2-one	30086-02-3	23.486	1089	1068	MS	419.49 ± 6.01 a	132.41 ± 7.80 b	123.51 ± 25.38 b
#35	Linalool	78-70-6	24.058	1097	1098	MS, RI	2880.55 ± 74.46 b	2816.48 ± 150.69 b	3251.05 ± 210.53 a
#36	3,7-Dimethylocta-1,5,7-trien-3-ol	29957-43-5	24.288	1100	1110	MS, RI	939.91 ± 4.51 b	318.63 ± 26.05 c	1073.64 ± 22.23 a
#37	Nonanal	124-19-6	24.465	1103	1102	MS, RI	489.33 ± 39.91 a	425.35 ± 5.87 b	461.09 ± 33.96 ab
#38	Isophorone	78-59-1	25.392	1119	1118	MS, RI	23.62 ± 1.28 b	38.18 ± 3.12 a	42.16 ± 9.23 a
#39	Caprylic acid methyl ester	111-11-5	25.941	1128	1120	MS, RI	17.07 ± 1.98	n.d.	n.d.
#40	(E,E)-Alloocimene	3016-19-1	26.115	1132	1140	MS, RI	62.49 ± 6.79 a	37.13 ± 5.51 b	n.d.
#41	(E)-3-Nonen-2-one	18402-83-0	26.709	1142	n.f.	MS	46.91 ± 2.85 a	46.19 ± 13.26 a	20.47 ± 5.08 b
#42	2,6,6-Trimethyl-2-cyclohexene-1, 4-dione	1125-21-9	26.967	1146	1139	MS, RI	68.98 ± 5.33 a	28.07 ± 4.49 b	33.00 ± 6.03 b
#43	1,2-Dimethoxybenzene	91-16-7	27.074	1148	1148	MS, RI	74.81 ± 2.36 b	115.53 ± 10.95 a	n.d.
#44	3,5-Dimethylphenol	108-68-9	27.227	1151	1169	MS, RI	38.63 ± 1.60	n.d.	n.d.
#45	4-(5-Methyl-2-furyl)butan-2-one	13679-56-6	27.344	1153	n.f.	MS	180.87 ± 3.95 b	243.6 ± 14.08 a	103.1 ± 19.15 c
#46	(2E,6Z)-2,6-Dodecadienal	21662-13-5	27.495	1156	1159	MS, RI	68.12 ± 8.32 a	37.77 ± 3.27 b	n.d.
#47	(E)-2-Nonenal	18829-56-6	27.902	1163	1166	MS, RI	70.57 ± 8.33 a	87.05 ± 11.76 a	62.19 ± 17.61 a
#48	Benzyl acetate	140-11-4	27.977	1164	1170	MS, RI	59.23 ± 3.92	n.d.	n.d.
#49	4-Ethylphenol	123-07-9	28.214	1168	1168	MS, RI	76.40 ± 2.37	n.d.	n.d.
#50	(E)-Linalool oxide (Pyran type)	14049-11-7	28.351	1171	1173	MS, RI	248.83 ± 12.67 a	74.68 ± 13.45 c	122.94 ± 10.50 b
#51	(Z)-Linalool oxide (Pyran type)	39028-58-5	28.623	1175	1179	MS, RI	746.95 ± 34.20 a	300.15 ± 47.15 b	371.54 ± 24.20 b
#52	4-Terpineol	562-74-3	28.816	1179	1179	MS, RI	452.31 ± 40.24 c	647.01 ± 33.61 b	765.63 ± 29.31 a
#53	4-Methylacetophenone	122-00-9	29.023	1182	1183	MS, RI	77.62 ± 2.19 a	47.41 ± 1.92 b	44.35 ± 10.13 b
#54	p-Cymen-8-ol	1197-01-9	29.186	1185	1184	MS, RI	135.64 ± 0.89 a	76.38 ± 6.29 b	n.d.
#55	Methyl salicylate	119-36-8	29.319	1187	1190	MS, RI	443.01 ± 28.76 a	78.90 ± 6.70 c	153.48 ± 12.78 b
#56	(R)-(+)-α-Terpineol	7785-53-7	29.563	1192	1195	MS, RI	1959.08 ± 139.25 c	3080.27 ± 212.35 b	3610.74 ± 150.39 a
#57	Safranal	116-26-7	29.684	1194	1221	MS, RI	398.54 ± 24.01 a	259.84 ± 38.55 b	306.08 ± 15.23 b
#58	Decyl aldehyde	112-31-2	30.166	1203	1207	MS, RI	233.90 ± 10.89 b	286.21 ± 10.84 b	398.55 ± 30.15 a
#59	3,5-Dimethylbenzaldehyde	5779-95-3	30.398	1209	1169	MS	32.30 ± 2.19 b	55.18 ± 3.41 a	n.d.
#60	β-Cyclocitral	432-25-7	30.57	1214	1224	MS, RI	310.10 ± 0.35 a	160.3 ± 27.82 b	22.28 ± 5.20 c
#61	Nerol	106-25-2	30.902	1222	1228	MS, RI	207.45 ± 6.97 a	193.13 ± 25.46 a	197.60 ± 10.69 a
#62	3,4-Dimethoxytoluene	494-99-5	31.46	1237	1230	MS, RI	167.26 ± 3.32 a	72.79 ± 17.33 b	n.d.
#63	(E)-Thujone	471-15-8	31.68	1242	1115	MS	n.d.	49.74 ± 4.15 b	66.16 ± 9.33 a
#64	Geraniol	106-24-1	31.966	1250	1277	MS, RI	458.96 ± 47.65 c	176.49 ± 20.43 b	607.89 ± 35.27 a
#65	(E)-2-Decenal	3913-81-3	32.403	1261	1263	MS, RI	n.d.	n.d.	147.32 ± 27.53
#66	Citral	5392-40-5	32.608	1266	n.f.	MS	n.d.	n.d.	60.68 ± 15.21
#67	4-Ethyl-2-methoxyphenol	2785-89-9	32.778	1271	1282	MS	297.02 ± 3.90	n.d.	n.d.
#68	1-Methylnaphthalene	90-12-0	33.534	1290	1297	MS, RI	40.27 ± 3.17 b	n.d.	54.62 ± 8.66 a
#69	2-Undecanone	112-12-9	33.587	1292	1291	MS, RI	46.74 ± 2.08 a	50.7 ± 5.64 a	n.d.
#70	(E,Z)-2,4-Decadienal	25152-83-4	33.643	1293	1293	MS, RI	17.28 ± 2.71 b	67.21 ± 1.37 a	n.d.
#71	Isopropyl salicylate	607-85-2	33.874	1299	n.f.	MS	n.d.	685.67 ± 24.29	n.d.
#72	1,2,3-Trimethoxybenzene	634-36-6	34.069	1305	1309	MS, RI	70.99 ± 6.01 b	113.59 ± 8.89 a	n.d.
#73	Theaspirane	36431-72-8	34.197	1309	1298	MS	82.80 ± 12.95 c	176.02 ± 18.47 a	119.21 ± 14.72 b
#74	4-Ethyl-1,2-dimethoxybenzene	5888-51-7	34.465	1318	n.f.	MS	107.06 ± 4.43 a	76.95 ± 9.87 b	47.13 ± 12.86 c
#75	3-Nonen-2-one	14309-57-0	35.067	1337	1135	MS, RI	46.69 ± 4.70 a	27.57 ± 1.64 b	n.d.
#76	Dehydro-ar-ionene	30364-38-6	35.492	1350	1355	MS, RI	n.d.	117.04 ± 15.08	n.d.
#77	γ-Nonalactone	104-61-0	35.687	1357	1358	MS, RI	46.09 ± 0.79 a	50.23 ± 17.9 a	n.d.
#78	2-Undecenal	2463-77-6	35.862	1362	1376	MS, RI	63.19 ± 10.37 a	n.d.	32.16 ± 3.57 b
#79	β-Damascenone	23726-93-4	36.273	1375	1384	MS	178.17 ± 13.77 a	115.38 ± 9.11 b	25.19 ± 4.70 c
#80	(Z)-Jasmone	488-10-8	36.697	1389	1396	MS, RI	81.79 ± 3.26 b	68.38 ± 17.05 b	173.82 ± 23.08 a
#81	6,10-Dimethyl-2-undecanone	1604-34-8	37.01	1399	n.f.	MS	62.59 ± 12.61	n.d.	n.d.
#82	Dodecanal	112-54-9	37.21	1405	1409	MS, RI	33.53 ± 1.02	n.d.	n.d.
#83	Dihydro-α-ionone	31499-72-6	37.278	1408	1406	MS, RI	39.26 ± 3.80 b	88.02 ± 1.21 a	n.d.
#84	α-Ionone	127-41-3	37.544	1417	1456	MS	576.76 ± 36.05 a	427.36 ± 20.75 b	256.29 ± 38.25 c
#85	Dihydro-β-ionone	17283-81-7	37.852	1427	1433	MS, RI	n.d.	175.77 ± 19.80	n.d.
#86	4-(2,2-Dimethyl-6-methylenecyclohexyl)butan-2-one	13720-12-2	37.92	1429	n.f.	MS	135.63 ± 15.20 a	n.d.	60.65 ± 6.07 b
#87	6,10-Dimethyl-5,9-undecadien-2-one	689-67-8	38.272	1441	1460	MS	480.24 ± 4.95 a	260.63 ± 6.19 b	272.99 ± 18.00 b
#88	Caryophyllene	87-44-5	38.417	1446	1418	MS	173.49 ± 28.06 b	40.21 ± 14.49 c	231.13 ± 15.42 a
#89	2,6-Di(tert-butyl)-4-hydroxy-4-methyl-2,5-cyclohexadien-1-one	10396-80-2	38.569	1451	1478	MS	24.53 ± 3.79 a	n.d.	20.04 ± 6.35 a
#90	(−)-Alloaromadendrenepurum	25246-27-9	38.651	1454	1460	MS, RI	n.d.	76.33 ± 10.90 a	52.49 ± 7.40 b
#91	4-Tert-butyl phenylacetone	81561-77-5	39.14	1471	n.f.	MS	223.22 ± 5.74 b	173.71 ± 9.07 c	264.15 ± 29.83 a
#92	β-Ionone	79-77-6	39.218	1473	1477	MS, RI	773.22 ± 30.16 a	605.41 ± 22.53 c	689.88 ± 19.29 b
#93	5,6-Epoxy-β-ionone	23267-57-4	39.326	1477	1455	MS	292.98 ± 15.55 a	231.77 ± 22.46 b	236.95 ± 15.66 b
#94	β-Cedrene	546-28-1	39.664	1488	1418	MS	n.d.	633.81 ± 8.90	n.d.
#95	1,5-Cyclodecadiene, 1,5-dimethyl	15423-57-1	39.86	1495	1555	MS	42.96 ± 6.29 a	n.d.	40.74 ± 9.13 a
#96	2,4-Ditert-butylphenol	96-76-4	40.045	1501	1512	MS, RI	496.77 ± 46.72 a	420.35 ± 22.51 b	555.81 ± 19.76 a
#97	(Z)-Calamenene	483-77-2	40.687	1523	1557	MS, RI	n.d.	238.64 ± 26.56	n.d.
#98	Dihydroactinidiolide	17092-92-1	40.732	1524	1493	MS	442.51 ± 17.6 b	741.08 ± 44.27 a	236.39 ± 10.27 c
#99	Nerolidol	40716-66-3	41.796	1560	1564	MS, RI	71.94 ± 15.49 a	25.78 ± 0.74 b	n.d.
#100	Spathulenol	6750-60-3	42.277	1576	1578	MS, RI	22.13 ± 2.09 b	90.98 ± 10.10 a	n.d.
#101	2,2,4-Trimethylpentanediol-1,3-diisobutyrate	6846-50-0	42.469	1583	1588	MS, RI	60.86 ± 7.74 a	74.44 ± 2.36 a	70.32 ± 18.72 a
#102	Caryophyllene oxide	1139-30-6	42.994	1600	1583	MS	32.70 ± 6.02 a	n.d.	29.05 ± 7.06 a
#103	Cedrol	77-53-2	43.173	1607	1601	MS, RI	44.11 ± 9.07 c	272.82 ± 13.70 a	137.99 ± 4.12 b
#104	Tridecane aldehyde	10486-19-8	43.376	1614	1518	MS	25.49 ± 6.28 b	76.41 ± 10.02 a	n.d.
#105	Epiglobulol	88728-58-9	44.144	1641	1564	MS	44.77 ± 7.36	n.d.	n.d.
#106	α-Cadinol	481-34-5	44.193	1643	1652	MS, RI	27.04 ± 4.10 a	n.d.	42.89 ± 13.72 a
#107	Isopropyl myristate	110-27-0	49.027	1822	1812	MS, RI	29.76 ± 3.21 a	36.24 ± 1.03 a	24.35 ± 10.23 a
#108	Hexahydrofarnesyl acetone	502-69-2	49.394	1840	1801	MS	111.98 ± 26.49 b	388.84 ± 30.93 a	100.39 ± 4.34 b
#109	Diisobutyl phthalate	84-69-5	49.739	1856	n.f.	MS	29.34 ± 2.32 a	n.d.	25.21 ± 0.67 b
#110	Dibutyl phthalate	84-74-2	51.419	1951	n.f.	MS	n.d.	54.95 ± 5.88 a	25.21 ± 4.37 b
#111	(Z)-7-Hexadecenal	56797-40-1	51.758	1972	1798	MS	17.16 ± 1.67 b	92.90 ± 4.58 a	10.68 ± 3.01 b
#112	Phytol	150-86-7	53.534	2104	2096	MS, RI	6.47 ± 2.44 b	57.82 ± 0.79 a	6.63 ± 1.41 b

Note: ^a^ Rt: Compounds were shown according to their order of appearance in the chromatogram on the DB-5MS column; ^b^ method of identification: RI, retention index; MS, identified by mass spectra; ^c^ in the same row, different letters indicate significant differences (*p* < 0.05); ^d^ n.d.: The compounds not detected in the sample; ^e^ n.f.: RI not found in the literature.

**Table 5:** The contents of the main non-volatile compounds of Tuo teas in the different grades.

Compounds ^a^	Content ^b^			VIP
	SG	1G	2G	
Water extract (%)	41.26 ± 0.06 a	39.38 ± 0.69 ab	37.99 ± 2.13 b	0.8091
Tea polyphenols (%)	19.07 ± 0.70 a	19.32 ± 1.23 a	17.56 ± 1.43 a	0.7911
Soluble sugars (%)	3.86 ± 0.13 a	4.22 ± 0.02 a	4.30 ± 0.36 a	0.7719
CAF (mg/g)	44.59 ± 1.62 a	47.29 ± 2.00 a	46.85 ± 3.25 a	0.7079
C (mg/g)	7.11 ± 0.28 a	5.27 ± 0.22 b	6.77 ± 0.43 a	1.3715
CG (mg/g)	0.86 ± 0.08 b	0.93 ± 0.05 b	1.20 ± 0.05 a	1.0486
EC (mg/g)	11.26 ± 0.36 a	9.76 ± 0.22 b	10.34 ± 0.83 ab	1.0583
ECG (mg/g)	34.65 ± 1.72 a	34.51 ± 1.45 a	33.89 ± 3.87 a	0.3467
EGC (mg/g)	36.76 ± 1.40 a	28.74 ± 1.67 b	30.01 ± 2.77 b	1.0816
EGCG (mg/g)	52.71 ± 2.78 a	50.30 ± 2.69 a	39.93 ± 2.94 b	1.0082
GC (mg/g)	24.66 ± 1.34 b	32.42 ± 0.56 a	29.51 ± 2.78 a	1.1897
GCG (mg/g)	1.01 ± 0.56 a	0.73 ± 0.03 a	1.00 ± 0.09 a	0.7236
Non-galloylated catechins (mg/g)	79.80 ± 3.38 a	76.21 ± 2.50 a	76.64 ± 5.15 a	0.6016
Ester catechins (mg/g)	89.23 ± 4.09 a	86.47 ± 4.14 a	76.02 ± 6.81 b	0.8797
Total catechins (mg/g)	169.03 ± 7.46 a	162.68 ± 6.59 a	152.66 ± 11.75 a	0.7298
GA (mg/g)	2.50 ± 0.08 c	3.74 ± 0.07 a	3.22 ± 0.17 b	1.3017
Ala (mg/g)	0.42 ± 0.01 a	0.42 ± 0.01 a	0.41 ± 0.00 a	0.6313
Arg (mg/g)	0.40 ± 0.01 b	0.55 ± 0.01 a	0.38 ± 0.01 b	1.4461
Asn (mg/g)	1.77 ± 0.01 a	1.50 ± 0.00 c	1.65 ± 0.02 b	1.3649
Asp (mg/g)	0.22 ± 0.02 a	0.19 ± 0.05 a	0.17 ± 0.04 a	0.5558
Cys (mg/g)	0.59 ± 0.03 b	0.73 ± 0.15 ab	0.80 ± 0.05 a	0.7922
GABA (mg/g)	0.22 ± 0.00 b	0.56 ± 0.08 a	0.18 ± 0.02 b	1.4196
Gln (mg/g)	1.61 ± 0.11 a	1.48 ± 0.11 a	1.22 ± 0.08 b	0.9386
Glu (mg/g)	0.55 ± 0.01 a	0.37 ± 0.02 b	0.41 ± 0.04 b	1.1778
His (mg/g)	0.47 ± 0.01 a	0.42 ± 0.01 b	0.38 ± 0.02 c	0.9745
Ile (mg/g)	0.32 ± 0.01 b	0.34 ± 0.01 b	0.39 ± 0.02 a	1.0221
Leu (mg/g)	0.40 ± 0.00 b	0.42 ± 0.01 a	0.35 ± 0.02 c	1.2514
Lys (mg/g)	4.87 ± 0.11 a	4.83 ± 0.31 a	4.94 ± 0.25 a	0.3314
Phe (mg/g)	0.20 ± 0.01 b	0.23 ± 0.00 a	0.17 ± 0.01 c	1.3057
Pro (mg/g)	0.67 ± 0.00 a	0.67 ± 0.00 a	0.66 ± 0.00 b	1.0283
Ser (mg/g)	0.04 ± 0.00 b	0.030 ± 0.02 b	0.08 ± 0.00 a	1.134
Thea (mg/g)	6.56 ± 0.11 a	5.89 ± 0.09 b	5.83 ± 0.18 b	1.0518
Thr (mg/g)	0.43 ± 0.02 a	0.38 ± 0.01 b	0.36 ± 0.01 c	0.9929
Trp (mg/g)	1.60 ± 0.06 a	1.66 ± 0.02 a	1.47 ± 0.07 b	1.1316
Tyr (mg/g)	0.59 ± 0.02 a	0.57 ± 0.00 b	0.55 ± 0.01 c	0.9596
Val (mg/g)	0.48 ± 0.01 b	0.47 ± 0.02 b	0.54 ± 0.02 a	1.1135
Total free amino acids (mg/g)	22.42 ± 0.31 a	21.70 ± 0.71 ab	20.93 ± 0.10 b	0.8744
Kae (mg/g)	0.0012 ± 0.0001 b	0.0010 ± 0.0001 b	0.0015 ± 0.0001 a	1.1929
Kae-gluc (mg/g)	0.0307 ± 0.0040 b	0.0376 ± 0.0007 a	0.0381 ± 0.0004 a	0.9706
Kae-rut (mg/g)	0.0670 ± 0.0084 a	0.0753 ± 0.0020 a	0.0768 ± 0.0011 a	0.8163
Myr (mg/g)	0.0010 ± 0.0002 a	0.0012 ± 0.0006 a	0.0009 ± 0.0001 a	0.5697
Myr-rha (mg/g)	0.0124 ± 0.0021 c	0.0160 ± 0.0003 b	0.0188 ± 0.0004 a	0.9507
Que (mg/g)	0.0010 ± 0.0003 b	0.0004 ± 0.0002 c	0.0020 ± 0.0001 a	1.2478
Que-gala (mg/g)	0.0180 ± 0.0028 c	0.0217 ± 0.0009 b	0.0253 ± 0.0004 a	0.922
Que-glu (mg/g)	0.0841 ± 0.0123 b	0.1074 ± 0.0024 a	0.1117 ± 0.0015 a	0.9722
Que-rut (mg/g)	0.1048 ± 0.0121 b	0.1258 ± 0.0036 a	0.1228 ± 0.0018 a	1.0088
Vit (mg/g)	0.0036 ± 0.0010 b	0.0072 ± 0.0003 b	0.0137 ± 0.0031 a	0.9739
Vit-rha (mg/g)	0.0166 ± 0.0011 b	0.0185 ± 0.0005 a	0.0191 ± 0.0003 a	0.931
Total flavones and flavonol glycosides (mg/g)	0.3404 ± 0.0442 b	0.4120 ± 0.0091 a	0.4308 ± 0.0055 a	0.9382

Note: ^a^ Caffeine (CAF), (+)-catechin (C), (−)-catechin gallate (CG), (−)-epicatechin (EC), (−)-epicatechin gallate (ECG), (−)-epigallocatechin (EGC), (−)-epigallocatechin gallate (EGCG), (−)-gallocatechin (GC), (−)-gallocatechin gallate (GCG), gallic acid (GA), Alanine (Ala), Arginine (Arg), Asparagine (Asn), Aspartic acid (Asp), Cysteine (Cys), γ-aminobutyric acid (GABA), Glutamine (Gln), Glutamic acid (Glu), Histidine (His), Isoleucine (Ile), Leucine (Leu), Lysine (Lys), Phenylalanine (Phe), Proline (Pro), Serine (Ser), Theanine (Thea), Threonine (Thr), Tryptophan (Trp), Tyrosine (Tyr), Valine (Val), kaempferol (Kae), kaempferol 3-O-glucopyranoside (Kae-gluc), kaempferol 3-O-rutinoside (Kae-rut), myricetin (Myr), myricetin 3-O-rhamnoside (Myr-rha), Quercetin (Que), quercetin 3-O-galactoside (Que-gala), quercetin 3-O-glucoside (Que-glu), quercetin-3-β-D-rutinoside (Que-rut), vitexin (Vit) and vitexin-2″-O-rhamnoside (Vit-rha); ^b^ In the same row, different letters indicate significant differences (*p* < 0.05).

## Data Availability

The original contributions presented in the study are included in the article, further inquiries can be directed to the corresponding author.

## Supplementary Materials

The following supporting information can be downloaded at: https://www.mdpi.com/article/10.3390/foods13060865/s1, Figure S1: Electronic nose analysis.
