# Supplementary figures and images for: Evaluation of Chongqing Tuo Tea at Different Grades: An Integrated Approach by Artificial and Intelligent Sensory, Non-Volatile, and Volatile Compounds Analysis
Source: Foods. 2024 Mar 13;13(6):865. doi: 10.3390/foods13060865 (PMC10969936; doi:10.3390/foods13060865)

## Supplementary materials

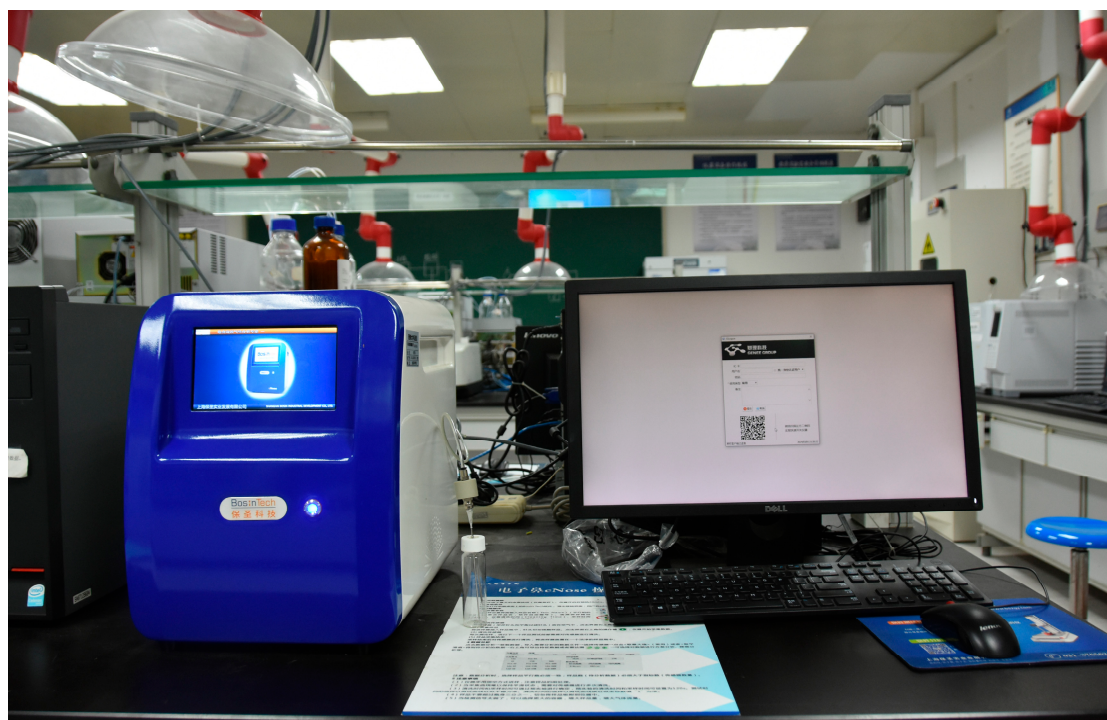

Figure S1. Electronic nose analysis

Supplement: Supplementary file 1 [file foods-13-00865-s001.zip › foods-2894099-supplementary.pdf]
